# A change from gonadotropin releasing hormone antagonist to gonadotropin releasing hormone agonist therapy does not affect the oncological outcomes in hormone sensitive prostate cancer

**DOI:** 10.1186/s12610-018-0074-2

**Published:** 2018-07-18

**Authors:** Jumpei Asakawa, Taro Iguchi, Satoshi Tamada, Sayaka Yasuda, Noriko Ninomiya, Minoru Kato, Takeshi Yamasaki, Tetusji Ohmachi, Tatsuya Nakatani

**Affiliations:** 1 Department of Urology, Osaka City University Graduate School of Medicine, 1-4-3 Asahi-machi, Abeno-ku, Osaka, 545-8585 Japan; 2 Department of Urology, Bell Land General Hospital, 500-3, Higashiyama, Naka-ku, Osaka, 599-8247 Japan

**Keywords:** Gonadotropin releasing hormone antagonists and inhibitors, Gonadotropin releasing hormone agonists, Prostate cancer, Degarelix, Castration-resistant

## Abstract

**Background:**

The aim of our retrospective study was to evaluate the 5-year survival and time to castration resistant prostate cancer in patients with hormone sensitive prostate cancer treated with the gonadotropin releasing hormone antagonist, degarelix. Another aim was to evaluate the effects of changing the treatment from degarelix to a gonadotropin releasing hormone agonist after achieving stable disease control, on the clinical and oncological outcomes.

**Results:**

Our analysis was based on the data of 108 patients with prostate cancer who were treated with degarelix. Of these, the treatment was changed from degarelix to a gonadotropin releasing hormone agonist in 57 patients (changed group), and the treatment with degarelix was continued in the other 51 (continued group). The overall 5-year survival was statistically superior in the changed (96.6%) group than that in the continued (74.1%) group (*p* = 0.006). The 5-year cancer-specific survival was also superior in the changed (100%) group than that in the continued (84.6%) group (*p* = 0.027). The average time to castration resistant prostate cancer was comparable in both the changed (43.3 months) and continued (35.2 months) groups (*p* = 0.117). Lower serum levels of prostate specific antigen and alkaline phosphatase were maintained after changing the therapy from degarelix to a gonadotropin releasing hormone agonist.

**Conclusions:**

Degarelix is effective in the treatment of prostate cancer. Degarelix therapy can also be safely changed to a gonadotropin releasing hormone agonist without any adverse clinical or oncological effects.

## Résumé

**Contexte:**

L’objectif de notre étude rétrospective était d’évaluer la survie à 5 ans et le délai de transition du cancer de la prostate hormono-sensible au cancer de la prostate résistant à la castration chez des patients porteurs d’un cancer de la prostate hormono-sensible qui étaient traités par un antagoniste de la gonadolibérine, le dégarélix. Un autre objectif était d’évaluer les effets sur les résultats cliniques et oncologiques du remplacement du dégarélix par un agoniste de la gonadolibérine après obtention d’un état stable et contrôlé.

**Résultats:**

Notre analyse repose sur les données de 108 patients atteints d’un cancer de la prostate traités par dégarélix. Parmi ceux-ci, le traitement par dégarélix a été remplacé par un agoniste de la gonadolibérine chez 57 (groupe modifié), et le traitement par dégarélix a été poursuivi chez les autres patients (groupe inchangé). La survie globale à 5 ans était statistiquement plus élevée pour le groupe modifié (96.6%) que pour le groupe inchangé (74,1%; *p* = 0.006). Les chances de survie cancer-spécifiques à 5 ans était également plus élevées pour le groupe modifié (100%) que pour le groupe inchangé (84,6%; *p* = 0.027). Le délai moyen de transition du cancer de la prostate hormono-sensible au cancer de la prostate résistant à la castration était comparable dans le groupe modifié (43,3 mois) et dans le groupe inchangé (35,2 mois). Des taux sériques plus bas d’antigène spécifique de la prostate et de phosphatase alcaline ont été maintenus après le remplacement du dégarélix par un agoniste de la gonadolibérine.

**Conclusions:**

Le dégarélix est. efficace dans le traitement du cancer de la prostate. Le traitement par dégarélix peut aussi être remplacé en toute sécurité par un agoniste de la gonadolibérine sans aucun effet clinique ou oncologique indésirable.

**Mots-clés:**

Antagonistes et inhibiteurs de la gonadolibérine, Agoniste de la gonadolibérine, Cancer de la prostate, Dégarélix, Résistant à la castration

## Background

Prostate cancer (PCa) is one of the most common cancers worldwide, with a survival rate that is relatively high in comparison with that of other cancers, partially due to the effectiveness of androgen deprivation therapy (ADT), even in patients with metastatic PCa. Ever since Huggins and Hodges [1] demonstrated that surgical castration resulted in significant clinical improvement in patients with advanced PCa, ADT has become the main treatment for PCa. The most commonly used agents for ADT are gonadotropin releasing hormone (GnRH) agonists. Although ADT can be effective in achieving testosterone levels comparable to those following castration (< 0.5 ng/ml), an initial testosterone surge is commonly observed, which can lead to an exacerbation of clinical symptoms, including urinary retention and aggravation of bone metastases [2]. To overcome this phenomenon, degarelix was developed as a novel GnRH antagonist, with evidence supporting its effectiveness in maintaining testosterone levels below the castration levels in PCa patients, as reported in the CS21 study [3]. Degarelix was approved by the Food and Drug Administration in 2008 and by the Pharmaceuticals and Medical Devices Agency of Japan in 2012. Degarelix blocks GnRH receptors in the pituitary gland, resulting in a rapid decrease in the production of testosterone, with suppression of testosterone to castrated levels that is normally achieved within 1–3 days [4, 5]. Additional analysis of the CS21 study data revealed no significant difference between the effects of GnRH antagonist and GnRH agonist on prostate specific antigen (PSA) progression [6]. However, in patients with a PSA level > 20 ng/ml, the use of GnRH antagonist significantly prolonged the time to recurrence of PSA elevation [6]. Degarelix also reduces the level of serum alkaline phosphatase (ALP) to a significantly greater extent in patients with bone metastases than does leuprorelin [7]. However, the effects of degarelix on androgen deprivation last for only 1 month; therefore, monthly administrations are required. The development of injection-site induration and pain are the main causes of treatment discontinuation with GnRH agonists. In contrast, GnRH antagonists are available in 1-, 3-, and 6-month depot formulations, and local reaction to subcutaneous injection of GnRH agonists is relatively rare, in comparison with degarelix. Therefore, it is common to switch from degarelix to a GnRH agonist once the testosterone levels are successfully reduced and stabilized. However, the possible effects of switching from degarelix to GnRH agonists on clinical and oncological outcomes in PCa have not been evaluated.

Hinotsu et al. reported a clinically acceptable progression-free survival and overall survival in patients treated with ADT for PCa [8]. However, in their study, a GnRH agonist was used for ADT and the time of transition from hormone sensitive prostate cancer (HSPC) to castration resistant prostate cancer (CRPC) was not investigated. To address these limitations, we retrospectively evaluated the overall survival and calculated the time of transition from HSPC to CRPC when treated with GnRH antagonist. We also evaluated the effects of switching from GnRH antagonist to GnRH agonist in PCa treatment on clinical and oncological outcomes.

## Methods

We conducted a retrospective cohort analysis of patients with histologically confirmed adenocarcinoma of the prostate, who underwent ADT by GnRH antagonist therapy at Osaka City University Hospital and Bell Land General Hospital. This study included 108 patients who were treated with degarelix, at an initial dose of 240 mg and maintenance dose of 80 mg. In 57 of these patients, degarelix was discontinued due to immediate achievement of decrease in PSA levels or due to adverse events of the drug, and a GnRH agonist (leuprorelin acetate or goserelin acetate) was used for maintenance therapy. Patients in whom degarelix was effective and who did not need switching to GnRH agonists continued degarelix. Adverse events due to degarelix were investigated monthly. In approximately all the patients (94.4%), bicalutamide (80 mg) was administered concomitantly with degarelix, and it was continued even after switching to GnRH agonists. Relevant characteristics of our patient groups are summarized in Table 1.

**Table 1:** Patients’ characteristics

		Degarelix (Continued group)		Conversion from degarelix to GnRH agonist (Changed group)		*p* value
Number of patients			51		57	
Follow up duration (months)	median (range)	19.4	(1.0–57.9)	38.8	(2.2–58.6)	< 0.001
Degarelix treatment duration (months)	median (range)	14	(1–52)	8	(1–39)	0.064
Age (years)	median (range)	73	(49–95)	75	(50–90)	0.572
T stage	T1	3	5.9%	1	1.8%	0.387
	T2	10	19.6%	18	31.6%	
	T3	24	47.1%	26	45.6%	
	T4	14	27.4%	12	21.1%	
N	N0	29	56.9%	31	54.4%	0.796
	N1	22	43.1%	26	45.6%	
M	M0	16	31.4%	23	40.4%	0.486
	M1	35	68.6%	34	59.6%	
Gleason score	6	1	2.0%	2	3.5%	0.389
	7	5	9.8%	6	10.5%	
	8	21	41.2%	24	42.1%	
	9	15	29.4%	15	26.3%	
	10	4	7.8%	5	8.8%	
	unknown	5	9.8%	5	8.8%	
Initial PSA (ng/ml)	Median (range)	145	(0.48–8072)	122	(5.62–9675)	0.669
PSA	PSA 0–4	1	2.0%	0	0.0%	0.415
	PSA4–10	5	9.8%	3	5.3%	
	PSA10–20	2	3.9%	5	8.8%	
	PSA > 20	43	84.3%	49	86.0%	
Treatment	CAB	48	94.1%	54	94.7%	0.889
	GnRH only	3	5.9%	3	5.3%	
Transition to CRPC	No	37	72.5%	38	66.7%	0.508
	Yes	14	27.5%	19	33.3%	
% PSA decrease by degarelix	median (range)	−99.4%	(6.6–100.0)	−99.8%	(47.2–100.0)	0.068

Abbreviations *CAB* combined androgen blockade, *CRPC* castration resistant prostate cancer, *GnRH* gonadotropin releasing hormone

The overall survival, cancer-specific survival, time to CRPC, serum PSA, and levels of testosterone and ALP were investigated. PSA progression was defined according to the Prostate Cancer Clinical Trials Working Group criteria as a 25% increase from the baseline value, with an absolute increase of 2 ng/mL after 12 weeks of treatment [9]. The declines in PSA and ALP levels from baseline following treatment with degarelix were calculated, with the changes in ALP levels analyzed only in patients with bone metastases. PSA and ALP levels were also compared before and after switching the treatment from degarelix to GnRH agonist. Again, ALP was analyzed only in patients with bone metastases. Testosterone levels were not routinely measured except to determine if below castration levels were maintained after switching to the GnRH agonist.

Differences in clinicopathological variables were compared between the two groups (the ‘continued’ and the ‘changed’ groups) by chi-squared analysis. The Kaplan-Meier method was used to compare the overall and cancer specific survival rate, and time to CRPC, with between-group differences in survival curves evaluated using log rank tests. The changes in PSA and ALP levels between the two groups were evaluated using a paired t-test. All *p*-values were two-sided, and a *p*-value < 0.05 was considered to be statistically significant. Statistical analysis was performed using Microsoft Excel®.

The access to the medical records of these patients was approved by the local research ethics committee at Osaka City University (approval number 4011).

## Results

The reasons to switch from degarelix to GnRH agonist in the 57 patients in the ‘changed group’ were as follows: injection site induration and pain (*n* = 12); allergic reaction to degarelix (*n* = 1); and immediate decrease in PSA (*n* = 44). Relevant characteristics were comparable between the two groups, except for the duration of follow-up (Table 1). Of note, compared with the patients on ADT for PCa in Japan [8], our study group included a higher proportion of cases with lymph node involvement and distal metastases. Additionally, the Gleason scores and initial PSA levels were relatively high in our study.

**Fig. 1:**
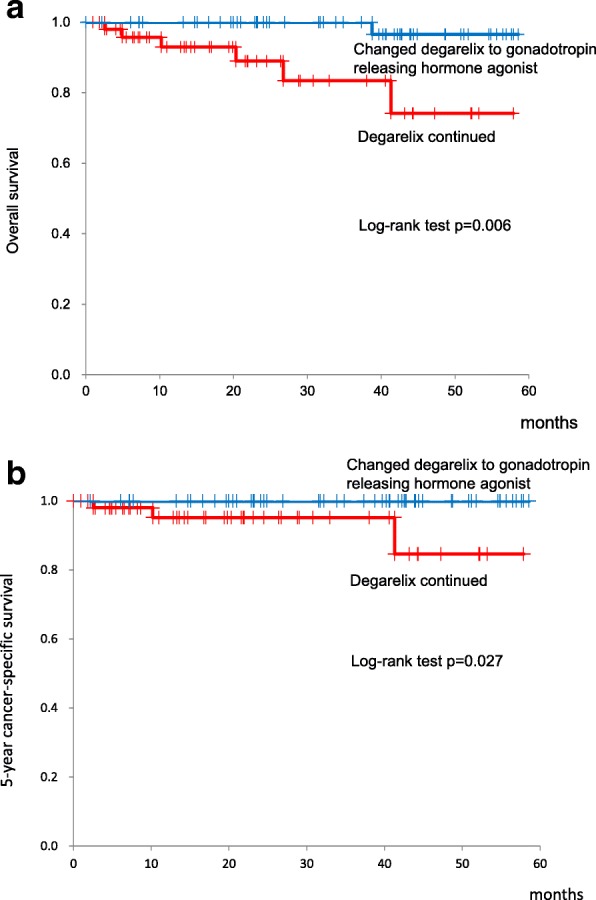
Overall survival (**a**) and cancer specific survival (**b**) in patients treated with degarelix and patients in whom degarelix was switched to gonadotropin releasing hormone agonist

**Fig. 2:**
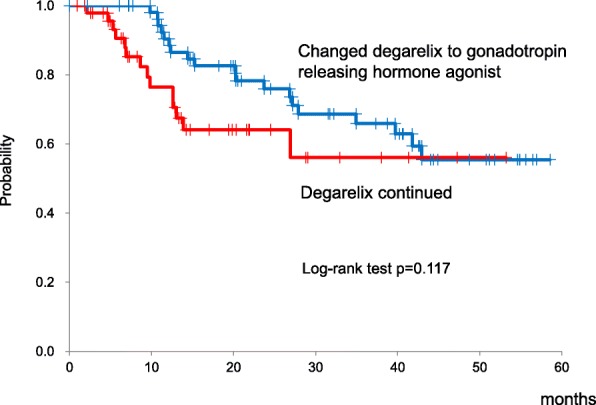
Time to castration resistant prostate cancer in patients treated with degarelix and patients in whom degarelix was switched to gonadotropin releasing hormone agonist

Of the 51 patients who continued degarelix (continued group), 3 patients died of PCa and 3 other patients died of a cerebrovascular accident, ischemic bowel disease, and unknown cause. Of the 57 patients in the changed group, only 1 patient died from acute myocardial infarction. Of the 108 patients who initially received degarelix, the 5-year overall survival rate, 5-year cancer-specific survival, and time to CRPC were 88.5, 95.0%, and 41.1 months (95% confidence interval (CI), 36.4–45.8 months), respectively. The overall survival was statistically superior in the ‘changed’ group in comparison with the ‘continued’ group, with a 5-year overall survival of 96.6 and 74.1%, respectively (*p* = 0.006) (Fig. 1a). The 5-year cancer-specific survival was also superior in the changed (100%) group compared with the continued (84.6%) group (*p* = 0.027) (Fig. 1b). The time to CRPC is shown in Fig. 2 for both groups, with no between-group differences identified: 35.2 months in the continued group and 43.3 months in the changed group (*p* = 0.117). The median percentage decrease in PSA level for all patients treated with degarelix was 99.7% (range, 0–100%), with maintenance of the lowered levels even after switching to GnRH agonists in the ‘changed’ group (Table 2). The levels of ALP in patients with bone metastases were also suppressed by degarelix (median decrease, 46.4%; range, 94.4% to − 144.4%), with the level of ALP remaining constant after switching to GnRH agonists in the ‘changed’ group (Table 2). Furthermore, the switch to GnRH agonists did not affect the serum testosterone levels; they were maintained below the castration levels in the changed group (0.09 ± 0.05 ng/ml).

**Table 2:** PSA and ALP change

	PSA before switch from GnRH antagonist to agonist	PSA after switch from GnRH antagonist to agonist	*p* value
PSA (ng/ml)	2.7 ± 8.8	2.0 ± 5.1	0.286
	ALP before switch from GnRH antagonist to agonist	ALP after switch from GnRH antagonist to agonist	
ALP (IU/L)	378 ± 545	387 ± 772	0.905

Abbreviations *PSA* prostate specific antigene, *ALP* alkaline phosphatase, *GnRH* gonadotropin releasing hormone

## Discussion

Ours is the first study to report that changing the treatment from a GnRH antagonist (degarelix) to a GnRH agonist did not affect the oncological outcomes in patients with hormone sensitive PCa. It is widely known that ADT is effective for the treatment of locally advanced PCa or metastatic PCa. As such, castration and GnRH agonist therapy remain the mainstream treatments for patients with advanced PCa. Results of the CS21 study [3] demonstrated that GnRH antagonists are effective in promptly reducing testosterone levels and suppressing the initial testosterone surge that is common in ADT. Therefore, results from the initial CS21 study with a 12-month follow-up, and its extended study to a 27.5-month follow-up [10], indicate that immediate disease control can be achieved with degarelix, a GnRH antagonist, which is comparable to that achieved with the use of GnRH agonists. However, long-term follow-up data on the clinical outcomes of degarelix use is not currently available. Furthermore, once stable disease status has been achieved, due to the negative effects of repeated monthly administration at the injection site, switching to GnRH agonists is common in patients with severe reactions to degarelix. The effects of this change in treatment on clinical outcomes have not been previously reported. These gaps in the current knowledge regarding the use of degarelix motivated us to conduct this study.

We report the first long-term survival data of degarelix. The 5-year overall and cancer-specific survival rates were high in both groups; 88.5% in the continued group and 95.0% in the changed group. Furthermore, considering the high Gleason score, serum PSA level, and high proportion of patients with metastatic PCa in our study group, degarelix in the treatment of PCa might be much more effective than expected. As a comparison, of the patients treated with ADT, Hinotsu et al. reported the following 5-year overall survival rates: 90% (stage II), 80% (stage III), and 60% (stage IV) [8].

It was obvious from this study that CRPC conversion took approximately 3–4 years, although it did not reach the median at the time of our data analysis. Few studies have reported on the time to CRPC in ADT. The ChemoHormonal Therapy Versus Androgen Ablation Randomized Trial for Extensive Disease in Prostate Cancer (CHAARTED) study by Sweeney et al. reported a median time to CRPC of 11.7 months in patients treated only with ADT [11]. Although baseline characteristics between our study cohort and the CHAARTED cohort were not equivalent, the differences in the time to CRPC might reflect the differences in the treatment of PCa between the two studies. In our study cohort, a majority of patients received antiandrogen drugs (bicalutamide 80 mg) in combination with degarelix. Akaza et al. reported a significant overall survival advantage favoring the use of combined androgen blockade (CAB) over GnRH agonist monotherapy (Cox regression analysis HR, 0.78) [12]. Similarly, Klotz et al. evaluated the efficacy of combining CAB therapy with bicalutamide 50 mg using validated statistical methodology to combine the Prostate Cancer Trialists’ Collaborative Group meta-analysis data [13] with data from a phase 3 trial of two CAB regimens (GnRH agonist plus bicalutamide 50 mg versus GnRH agonist plus flutamide) [14]. Their analysis demonstrated that the combination of CAB with bicalutamide 50 mg could reduce the risk of death by 20% compared with castration alone (HR, 0.80). In the CHAARTED study, the median overall survival was 13.6 months longer with ADT plus docetaxel than with ADT alone (57.6 months versus 44.0 months; HR for death in the combination group, 0.61; 95% CI, 0.47–0.80; *P* < 0.001). Recently, the addition of abiraterone acetate and prednisone to ADT has significantly increased the overall survival and radiographic progression-free survival in metastatic HSPC [15]. Considering these data, it is likely that the overall survival can be extended with the use of ADT plus other drugs compared with GnRH antagonist/agonist monotherapy.

As previously described, continuous administration of degarelix is troublesome due to the adverse events (skin reaction at the injection site) and the frequency of hospital visits as degarelix is only available in 1-month doses. Therefore, it is common practice to change the treatment from degarelix to a GnRH agonist once a stable PCa status has been achieved. In their evaluation of the endocrine assessment of the gonadal axis in patients whose treatment was changed from a GnRH antagonist to GnRH agonist, Miyazawa et al. identified a testosterone surge in 8.3% of their study patients, with this surge being mild and of very short duration [16]. Furthermore, Miyazawa et al. did not observe a significant change in PSA levels after changing to the GnRH agonist leuprolide, and no patients reported symptoms associated with a testosterone surge over the observation period. Based on their findings, they concluded that switching from degarelix to leuprolide (a GnRH agonist) appears to be a reasonable therapeutic option for patients with PCa. In our study cohort, the time to CRPC was comparable between patients in whom degarelix was continued and those in whom treatment was changed to a GnRH agonist, although the overall- and cancer-specific survival rates were statistically superior in those in whom treatment was changed. In agreement with Miyazawa et al., the change in ADT did not affect PSA and testosterone levels. Therefore, it is safe to conclude that switching from degarelix to a GnRH agonist is an effective treatment for PCa.

Assessment of ALP levels before and during PCa treatment might provide useful prognostic information, with ALP levels after 6 months of ADT being predictive of overall survival in patients with PCa [17, 18]. Fritz et al. reported that degarelix successfully reduced ALP levels and maintained ALP suppression, although leuprolide did not [7]. Our study did not support this finding, with levels of ALP reduction achieved with degarelix being maintained after the change to a GnRH agonist in patients with PCa and bone metastasis. This result also confirms that switching from degarelix to a GnRH agonist does not affect the disease control.

This study is a retrospective study and, thus, has certain limitations, including a selection bias which might have influenced our results. Our observation period was different for patients who continued degarelix and those who switched. The relatively short period of observation, with few cases of death, particularly in the changed group, may have influenced our group comparisons of overall- and cancer-specific survival. Both localized PCa and metastatic cases were included in our study group. Localized PCa should first be treated using a local approach, prostatectomy, or radiation therapy. Hormone therapy may be used in selected cases, based on the treatment complications, age, and comorbidities, among other factors. Further studies are needed to clarify the outcomes of ADT in patients who actually require hormonal therapy.

## Conclusions

Degarelix is effective in the treatment of prostate cancer. Degarelix therapy can also be safely changed to a GnRH agonist without any adverse clinical or oncological effects.
